# Clinical clues for suspecting wild-type transthyretin cardiac amyloidosis in patients with monoclonal gammopathy of undetermined significance: a case report

**DOI:** 10.1186/s43044-024-00499-x

**Published:** 2024-06-10

**Authors:** Tomoaki Haga, Takahiro Okumura, Yasuhiko Harada, Hiroaki Hiraiwa, Ryota Morimoto, Shinji Kaneko, Nagaaki Kato, Masanori Shinoda, Toyoaki Murohara

**Affiliations:** 1 Department of Cardiology, Nagoya University Graduate School of Medicine, 65, Tsurumai-cho, Showa-ku, Nagoya, Aichi 466-8560 Japan; 2 Department of Cardiology, Toyota Kosei Hospital, Toyota, Japan; 3 Department of Hematology, Toyota Kosei Hospital, Toyota, Japan; 4 Department of Medicine (Neurology and Rheumatology), Shinshu University School of Medicine, Matsumoto, Japan; 5 The Institute for Biomedical Sciences, Shinshu University, Matsumoto, Japan

**Keywords:** Transthyretin, Cardiac amyloidosis, Monoclonal gammopathy of undetermined significance

## Abstract

**Background:**

Myeloproliferative disorders, including monoclonal gammopathy of undetermined significance (MGUS), are often associated with amyloid light-chain (AL)-type cardiac amyloidosis (CA) but occasionally with wild-type transthyretin (ATTR) CA. In recent years, ATTR amyloidosis has attracted necessity for its reliable diagnosis with the addition of new treatments. Usually, both wild-type ATTR CA and AL-type CA present with marked cardiac hypertrophy, but renal dysfunction is milder in wild-type ATTR amyloidosis than in AL-type amyloidosis. Peripheral neurologic and autonomic symptoms such as numbness and dysesthesia are moderately present in AL-type amyloidosis, but less so in wild-type ATTR amyloidosis. Furthermore, the prognosis of ATTR-type amyloidosis is better than that of AL-type amyloidosis.

**Case presentation:**

A 72-year-old man with cardiac hypertrophy presented with New York Heart Association functional class III dyspnea and leg edema. He had no history of carpal tunnel syndrome. An electrocardiogram showed atrial fibrillation and low voltage. The N-terminal pro-B-type natriuretic peptide level was 3310 pg/mL, and troponin T was elevated to 0.073 ng/mL. However, the glomerular filtration rate was only slightly decreased at 69.0 mL/min/1.73 m^2^. The serum free light-chain assay revealed a significant increase in the kappa chain, with positive results in Bence Jones proteins and serum immunoelectrophoresis. Bone marrow examination confirmed the diagnosis of monoclonal gammopathy of undetermined significance (MGUS). AL-type amyloidosis associated with a myeloproliferative disorder was suspected, and the prognosis was initially predicted to be poor, classified as Mayo stage IV. Contrary to this prognosis, the patient showed a slow progression of heart failure. Further imaging modalities and cardiac tissue findings confirmed the diagnosis as transthyretin type amyloidosis, and a favorable prognosis was established with the use of tafamidis.

**Conclusions:**

MGUS occasionally coexists with wild-type ATTR CA. Scant autonomic symptoms, mild renal dysfunction, and slow progression of heart failure might be clues that the CA associated with the myeloproliferative disease is wild-type ATTR amyloidosis.

## Background

Amyloidosis is characterized by multiple organ dysfunction due to the deposition of β-sheet-structured amyloid fibrils [1]. Cardiac amyloidosis (CA) is a progressive infiltrative cardiomyopathy that presents with restrictive cardiomyopathy and conduction system diseases. The prognosis of CA is poor, although recent progress in therapeutic interventions has contributed to improved prognosis in patients with CA. The pathological typing of CA is important because therapeutic interventions and prognosis differ depending on the subtype of amyloidosis [2].

Myeloproliferative disorders, including monoclonal gammopathy of undetermined significance (MGUS), are often associated with amyloid light-chain (AL)-type CA but occasionally with wild-type amyloid transthyretin (ATTR)-CA. However, the older patients and patients in poor general condition tend to hesitate to undergo highly invasive advanced examinations. In addition, currently in the world, only a limited number of institutions can perform immunostaining, and even fewer institutions can stain all anti-transthyretin (TTR), anti-kappa, and anti-lambda antibodies. Thus, if a biopsy is not available or if the biopsy specimen stains positive for Congo red or direct fast scarlet and presents with monoclonal gammopathy, the patient is sometimes diagnosed as having AL-type amyloidosis. This diagnostic dilemma may lead to potential concerns that should be addressed. Here, we present a case of wild-type ATTR CA with MGUS, which was suspected to be a non-AL-type due to some noteworthy clinical clues.

## Case presentation

A 72-year-old male presented with dyspnea and leg edema. The patient previously had four catheter ablations for atrial fibrillation, but there was persistence of irregular rhythm. There was no history of spinal canal stenosis or carpal tunnel syndrome. Additionally, orthostatic hypotension and numbness symptoms in the extremities were not evident. The blood pressure and heart rate were 123/73 mmHg and 92 beats per minute, respectively. Oxygen saturation was 96% in room air. Physical examination revealed leg edema and jugular vein distention.

**Fig. 1:**
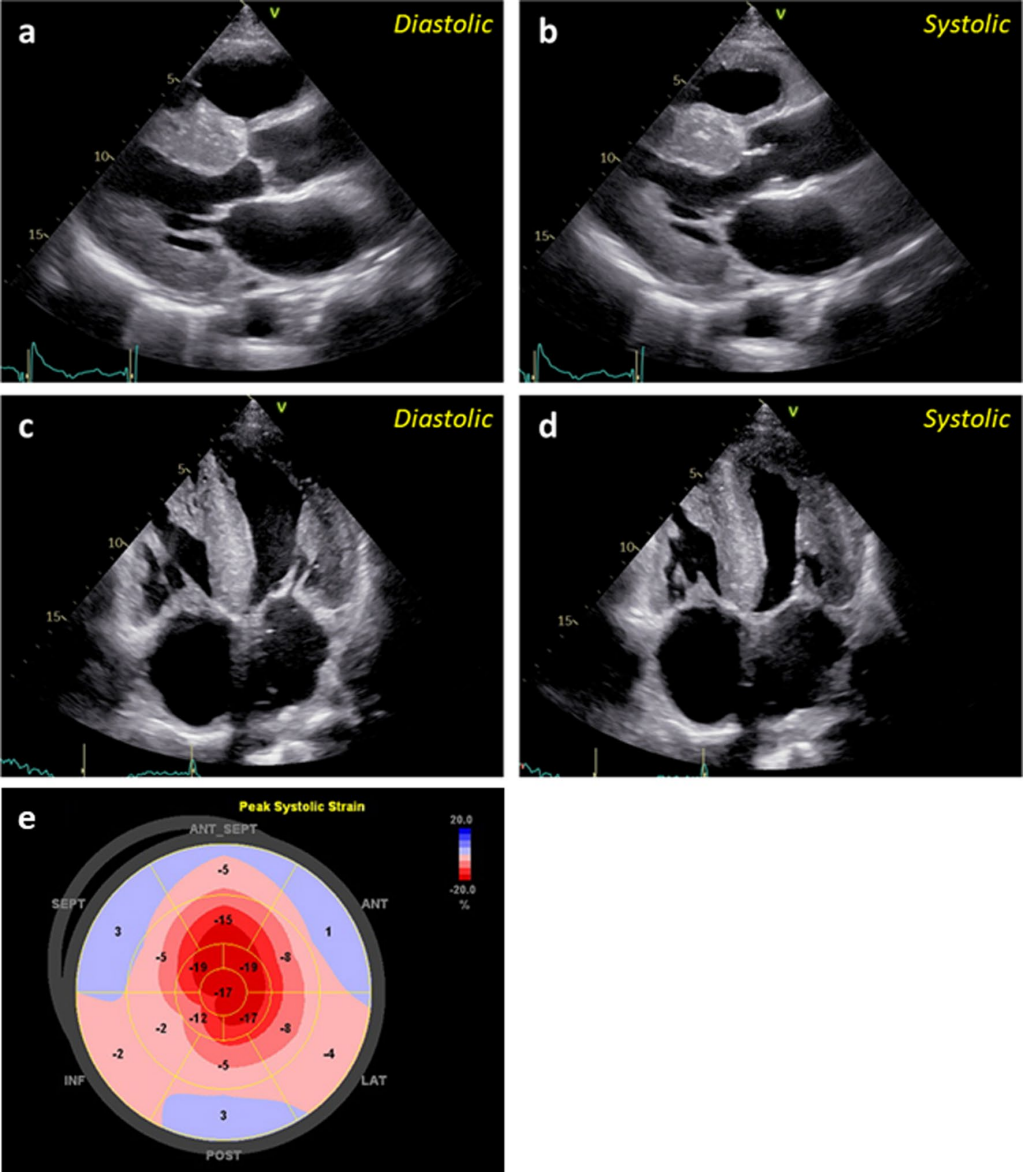
Diffuse cardiac hypertrophy and apical sparing pattern on transthoracic echocardiography. Symmetric left ventricular hypertrophy and mild systolic dysfunction are observed in the parasternal long axis view (**a**,** b**) and four-chamber view (**c**,** d**). Two-dimensional speckle-tracking echocardiography shows a reduced longitudinal strain in the basal segments and preserved longitudinal strain in the apical segments of the left ventricular myocardium (apical sparing) (**e**)

Chest radiography showed cardiothoracic enlargement with a cardiothoracic ratio of 0.56 and mild pulmonary congestion. Electrocardiogram showed atrial fibrillation rhythm, low QRS amplitude in limb leads, poor R progression in V_1_ to V_3_ leads, and ST depression in V_5_ and V_6_ leads. Symmetric left ventricular hypertrophy and mild systolic dysfunction with a left ventricular ejection fraction of 40.0% were observed on echocardiography (Fig. 1). Echocardiographic apical sparing strain patterns were also documented.

N-terminal pro-B-type natriuretic peptide (NT-proBNP) and troponin T levels were 3,310 pg/mL (normal: < 125 pg/mL) and 0.073 ng/mL (normal: < 0.015 ng/mL), respectively. The estimated glomerular filtration rate of the patient was 69.0 mL/min/1.73 m^2^ and urine protein excretion of the patient was 0.16 g/day; thus, the staging of chronic kidney disease was G2A2. The patient was treated with loop diuretics and tolvaptan for symptoms of heart failure (New York Heart Association (NYHA) functional class III). In the serum free light-chain assay, the kappa/lambda ratio was 23.4 (374 mg/L, (normal: 3–19 mg/mL) / 16 mg/L (normal: 6–26 mg/mL)) and different chain level was 358 mg/L. In addition, the Bence Jones proteins and serum immunoelectrophoresis were positive. According to Mayo AL stage 2012 [3], the patient was classified as stage IV, and the 6-month prognosis was estimated to be approximately 50%.

For a more rapid and accurate definitive diagnosis, a pathological evaluation by endomyocardial biopsy, which is invasive but has high detection accuracy, was favored. Coronary angiography revealed no evidence of ischemic stenosis. The pathological findings of the myocardial samples taken from the right ventricle showed positive Congo red staining, which provided a definitive diagnosis of CA. Bone marrow aspiration revealed 7.8% plasmacytes, which was consistent with the diagnosis of MGUS. The proportion of plasmacytes doubled in 6 months. In addition, the hemoglobin level decreased from 10.6 to 9.1 g/dL. Initially, AL-type CA associated with myeloproliferative disorders was clinically suspected; however, the scarcity of autonomic symptoms, mild renal dysfunction, and slow progression of heart failure led us to re-examine the possibility of non-AL CA.

**Fig. 2:**
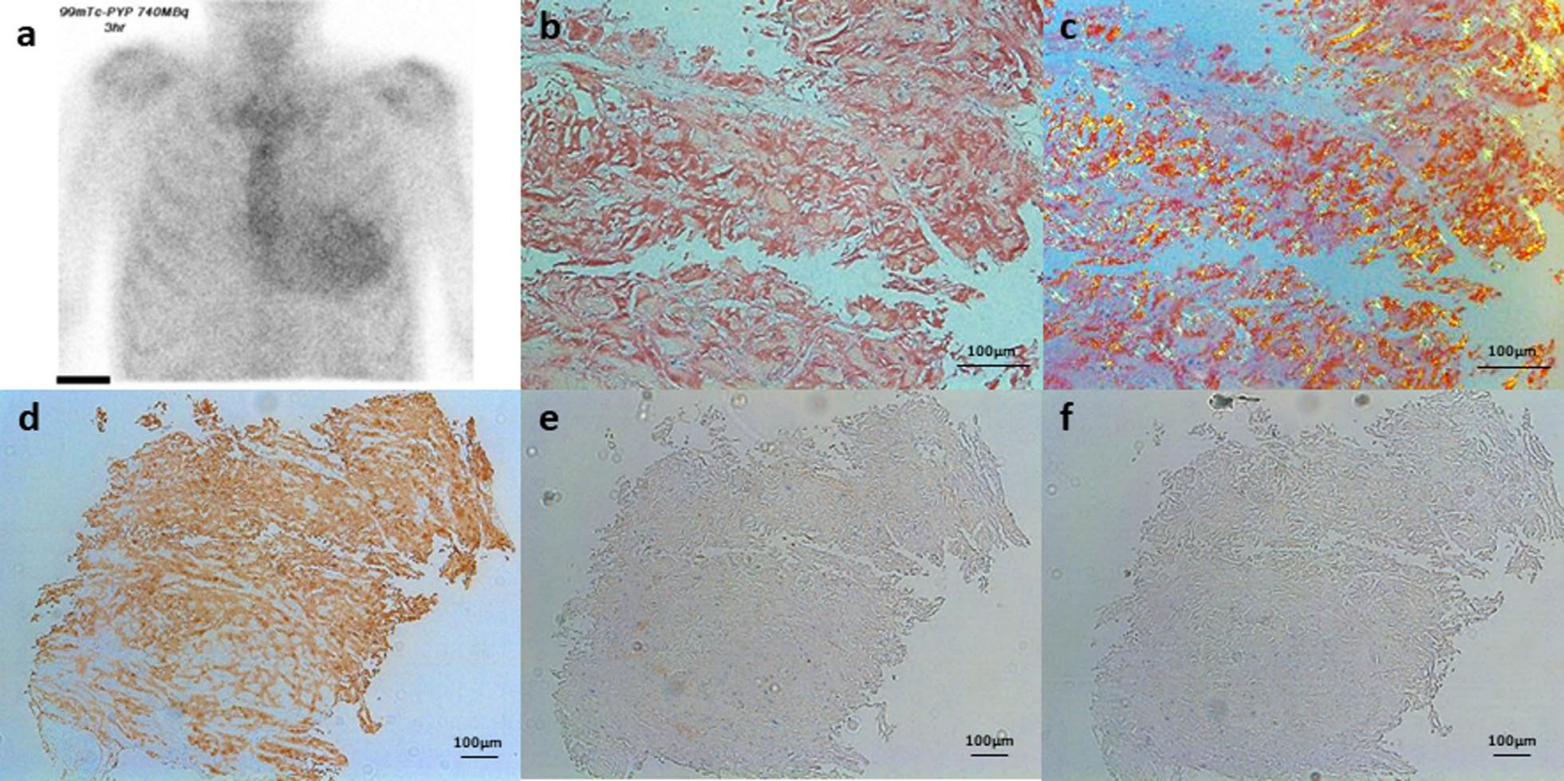
Increased uptake into the myocardium in ^99m^Technetium pyrophosphate (Tc-PYP) scintigram and histological findings of endomyocardial biopsy samples. Myocardial uptake of ^99m^Tc-PYP was greater than bone uptake, which was classified as Grade 3 (**a**). Congo red-positive amyloid deposits in the myocardium (**b**). Apple-green birefringence under polarized light (**c**). Myocardial amyloid deposits were immunologically stained with anti-transthyretin (**d**) but not with anti-kappa (**e**) and anti-AA (**f**)

^99m^Technetium and pyrophosphate (Tc-PYP) scintigraphy revealed grade 3 uptake of Tc-PYP in the myocardium (Fig. 2a). Although ATTR might be deposited in the myocardium, we were concerned that MGUS could potentially progress to multiple myeloma, which might be associated with the advanced stage of AL-type CA in the near future. Hence, chemotherapy with bortezomib and dexamethasone was administered. Immunostaining showed that the myocardium was stained with anti-TTR, but not anti-kappa, anti-lambda, or anti-AA (Fig. 2b–f). Genetic testing revealed no mutations in TTR-related genes. Therefore, the final diagnosis was wild-type ATTR CA with MGUS, and the patient was treated with 80 mg of oral tafamidis per day. Three years after starting treatment, NT-proBNP remained unchanged at 3,131 pg/mL and left ventricular ejection fraction at 40%, but he experienced only one heart failure hospitalization and his symptoms improved to NYHA functional class II.

## Discussion

This case was a wild-type ATTR CA with myeloproliferative disorder mimicking AL-type CA. The prevalence of MGUS is reported to be 5.3% in the general population aged over 70 years [4]. MGUS is considered a precursor to multiple myeloma, which is often associated with AL-type amyloidosis. Consequently, when biopsy or immunostaining is not available, there is concern that cases with an abnormal κ/λ ratio will be treated as AL-type CA. However, 10–18% of patients with MGUS-associated CA have wild-type ATTR amyloidosis and 23–39% of patients with ATTR wild-type amyloidosis have coexisting MGUS [5, 6]. Unfortunately, these findings are not yet widely understood. In recent years, ATTR amyloidosis has attracted necessity for its reliable diagnosis with the addition of new treatments such as tafamidis, patisiran, and vutrisiran [7].

Both AL-type amyloidosis and wild-type ATTR CA present with marked cardiac hypertrophy, but renal dysfunction is reported to be milder in wild-type ATTR amyloidosis than in AL-type amyloidosis (Table 1). Peripheral neurologic and autonomic symptoms such as numbness and dysesthesia are moderately present in AL-type amyloidosis, but less so in wild-type ATTR amyloidosis. Furthermore, the prognosis of ATTR-type amyloidosis, in particular wild type, is reported to be better than that of AL-type amyloidosis.

**Table 1:** Amyloidosis types and severity of lesions observed in each organ

Organ	AL-type	ATTR-type	
		Hereditary	Wild-type
Heart	***	**	***
Kidney	***	**	–
Autonomic nerves	**	***	–
Peripheral nerves	**	***	*
Gastrointestinal tract	**	***	–
Liver	**	–	–
Eye	*	**	–

*AL* Amyloid light-chain, *ATTR* Amyloid transthyretin

*Mild

**Moderate

***Severe

In this case, peripheral neurologic and autonomic symptoms were scant, and renal dysfunction was mild. In addition, the progression of heart failure was slower than initially expected and the clinical course was too long for AL-type amyloidosis based on Mayo AL stage 2012 [3]. These findings may be clues that the type of amyloidosis associated with the myeloproliferative disease is non-AL (mostly ATTR)-type rather than AL-type.

Neither lambda nor kappa light-chain amyloid deposition were pathologically observed, but the increase in plasmacytes in the bone marrow and the gradual decrease in hemoglobin levels indicated that MGUS was likely to progress to multiple myeloma. Due to the potential development of AL-type amyloidosis with poor prognosis, early chemotherapy was administered to reduce organ dysfunction. However, the advantages and disadvantages of front-loaded treatment are debatable. Furthermore, the type of amyloid that accumulates in the myocardium of MGUS patients with concomitant ATTR-type CA as they progress to multiple myeloma is unknown. For appropriate treatment, close monitoring and comprehensive pathological examination are recommended.

## Conclusions

MGUS occasionally coexists with wild-type ATTR CA. Scant autonomic symptoms, mild renal dysfunction, and slow progression of heart failure might be clues that the CA associated with the myeloproliferative disease is wild-type ATTR amyloidosis.

## Data Availability

Not applicable.

## Abbreviations

CA: Cardiac amyloidosis
MGUS: Monoclonal gammopathy of undetermined significance
AL: Amyloid light-chain
ATTR: Amyloid transthyretin
TTR: Transthyretin
NT-proBNP: N-terminal pro-B-type natriuretic peptide
NYHA: New York Heart Association
Tc-PYP: Technetium and pyrophosphate
